# *In vitro* antimicrobial activity of ozonated oil in liposome eyedrop against multidrug-resistant bacteria

**DOI:** 10.1515/med-2022-0495

**Published:** 2022-06-07

**Authors:** Giuseppe Grandi, Rossana Cavallo, Elisa Zanotto, Raffaella Cipriani, Claudio Panico, Romolo Protti, Giovanni Scapagnini, Sergio Davinelli, Ciro Costagliola

**Affiliations:** Microbiology and Virology Unit, University Hospital Città della Salute e della Scienza di Torino, Turin, 10126, Italy; Microbiology and Virology Unit, Amedeo di Savoia Hospital, Turin, 10149, Italy; Ophthalmology Unit, Humanitas Gradenigo Hospital, Turin, 10153, Italy; Ophthalmic Unit, Hospital San Biagio, Domodossola, 28845, Italy; Department of Medicine and Health Sciences “V. Tiberio”, University of Molise, Via V. De Sanctis, s.n.c., Campobasso 86100, Italy

**Keywords:** *in vitro* antimicrobial activity, ozonated oil, bacteria, ocular infections

## Abstract

Bacteria are the most common causative agents of ocular infections. Treatment with topical broad-spectrum antibiotics is recommended in severe cases. However, antibiotic resistance has become a major concern in recent years, although antibiotics are generally effective in treating ocular infections. Antibacterial compound screening is performed to identify alternative therapeutic options to antibiotics. The aim of this study was to assess the *in vitro* antimicrobial activity of an ophthalmic solution containing ozonated oil. Strains of bacterial species with a multidrug resistance profile, which are responsible for a large proportion of ocular infections, were isolated and selected from different biological samples. The bacterial isolates were cultured, and ozonated oil was used to evaluate the inhibition zones at different time points. The treatment exhibited antibacterial activity against all the tested species. The effect was lower against the strains of *Pseudomonas aeruginosa* and more evident against *Staphylococcus* and *Streptococcus* spp. Our results suggest that the administration of ozonated oil may be a candidate agent to treat some infections of the ocular surface with a potential role in antimicrobial prophylaxis.

## 1 Introduction

Like most parts of the body, the eyelids, conjunctiva, and corneal surface are colonized by resident bacteria. Imbalances in these microorganisms can trigger the onset of ocular infections, such as conjunctivitis, keratitis, and endophthalmitis [1,2]. This can be often attributed to external factors, such as the use of contact lenses, trauma, or surgical operations, or to patient-related factors, such as age, ocular dryness, or chronic obstruction of the nasolacrimal duct [3,4].

The eye is protected by the body’s innate and adaptive immunity; surface IgA and large amounts of antibacterial compounds, such as lysozyme and lactoferrin, are present in tears. However, the presence of these antibacterial compounds alone is not enough to prevent the development of infection, and subsequent inflammation and scarring do not heal very easily [5,6,7,8]. Severe eye infections require immediate management because they can damage the ocular tissues, leading to visual impairment and blindness. Although these conditions are mostly managed empirically, optimal treatment requires knowledge of the specific etiology of the condition. In western countries, the main causes of ocular infections are Gram-positive bacteria, including *Staphylococcus aureus*, coagulase-negative Staphylococci (CoNS), *Streptococcus* spp., and gram-negative bacteria, particularly *Pseudomonas aeruginosa* [9,10].

Conjunctivitis in adults is frequently caused by *Staphylococcus* spp., *Streptococcus* spp., *P. aeruginosa*, *Klebsiella pneumoniae,* and *Pasteurellae* spp., whereas *Haemophilus influenzae*, *Streptococcus pneumoniae,* and *Moraxella* spp. are more frequently responsible for the condition in children [11,12,13]. *S. aureus*, CoNS, and *Streptococcus* spp. are the most important pathogens responsible for keratitis. Meanwhile, *P. aeruginosa* is predominant in contact lens wearers and in patients who have experienced ocular trauma [14,15,16,17,18]. Endophthalmitis, occurring after cataract surgery or after intravitreal injection, is most commonly caused by CoNS, *S. aureus*, *Bacillus* spp., or *P. aeruginosa* [19,20,21].

Although antibiotics are generally effective in treating ocular infections, antibiotic resistance has become a widespread problem. This is largely due to the widespread and unwarranted use of broad-spectrum antibiotics in both systemic and topical infections, coupled with an inappropriate duration of treatment [22].

To prevent postoperative infections, it is important to reduce the bacterial load on the ocular surface through the use of antibiotics. However, if this use is not selective and well managed, there is a risk for multidrug resistance development [23,24,25].

The latest Antibiotic Resistance Monitoring in Ocular Microorganisms (ARMOR) study, published in 2018, covers 4829 bacterial isolates from ocular infections, collected from 87 centers across 40 states, in the United States, from January 2009 to December 2016, including *S. aureus*, CoNS, *S. pneumoniae*, *H. influenzae*, and *P. aeruginosa* [26]. This study demonstrated geographic variation in resistance rates among ocular isolates. Moreover, methicillin-resistant strains of both *S. aureus* and CoNS were highly resistant to fluoroquinolones, aminoglycosides, and macrolides. Strains of *S. pneumoniae* were found to be sensitive to all the antibiotics tested, and strains of *P. aeruginosa* exhibited low levels of resistance, particularly toward ciprofloxacin. These data highlight the need to re-evaluate and implement the guidelines for antibiotic treatment not only to prevent the emergence of drug resistance in pathogens but also to highlight the need to develop new drugs with high efficacy, low toxicity, and low resistance potential [27].

Therefore, research has begun to focus on molecules with antimicrobial and antiseptic potentials that are structurally different from conventional antibiotics. One such molecule is Ozone, which is a powerful oxidizing agent known for its antiseptic and anti-inflammatory properties. This molecule releases free oxygen radicals that facilitate the formation of hydrogen peroxide and lipid peroxidation products that are responsible for bacterial lysis and cell death. The introduction of ozonated agents has been advocated on the basis of efficacy against all microorganisms, as well as the lack of induction of antibiotic resistance [28]. Over the last few years, several therapeutic protocols using ozone have been developed to treat dental and skin infections. In these protocols, ozone is used in the following three different forms: gaseous ozone, ozonated water, and ozonated oil [29,30,31,32,33]. Several studies have evaluated the efficacy of ozonated oil formulations against microorganisms that are normally responsible for ocular infections [34,35,36]. The aim of this study was to evaluate the antibacterial activity of a liposomal ozonated oil solution for ophthalmic use against multidrug-resistant (MDR) bacterial strains.

## 2 Methods

From the bacterial strain library of the Comprehensive Structure of Microbiology and Virology Unit, University “Città della Salute e della Scienza,” Turin, Italy, 60 microorganisms with MDR profiles (bacteria resistant to at least three classes of antibiotics, including fluoroquinolones), previously isolated from different biological materials, were selected. These included (1) 20 strains of *P. aeruginosa* (PA MDR); (2) 20 strains of methicillin-resistant *S. aureus* (MRSA); and (3) 20 strains of methicillin-resistant *S. epidermidis* (MRSE). In addition, ten strains of *Streptococcus* spp. (five *S. pneumoniae* strains, three *S. agalactiae* strains, and two *S. pyogenes* strains) were analyzed even if they showed no significant resistance to antibiotics because they are widely represented in ocular infections. The antimicrobial susceptibility was determined by Kirby-Bauer antibiogram method. After reviving the microorganisms, which were frozen at −20°C, a suspension of the bacterial cultures with a turbidity of 0.5 McFarland was prepared. McFarland standards were used to prepare bacterial suspensions to a specified turbidity. In the Kirby-Bauer susceptibility test protocol, the bacterial suspension of the organism to be tested is equivalent to the 0.5 McFarland standard. MRSE, MRSA, and PA MDR cultures were transferred onto Mueller-Hinton agar (BD Diagnostics, Sparks, MD) plates using a spatula, while other *Streptococcus* spp. strains were transferred onto Mueller Hinton fastidious (BD Diagnostics, Sparks, MD) agar plates. Liposomal ozonated oil eye drops (Ozodrop, FB Vision, Italy), composed of ozonated liposomal sunflower oil (LipozonEye^®^, 10.5%) plus hypromellose (0.2%) and polyhexamethylene biguanide; sunflower ozonated oil, and hypromellose (hydroxypropyl methylcellulose), were deposited at the center of the plate at a final volume of 75 μL. Zone of inhibition was observed after incubation at 37°C for 2, 4, 6, 8, and 24 h. *Streptococcus* spp. strains were incubated at 37°C in a 5% CO_2_-enriched atmosphere. Descriptive statistical analysis, including percentages to characterize data, was performed using Microsoft Excel 2013 (Microsoft Corp.; Redmond, WA, USA). All experiments were performed in triplicate.

## 3 Results

**Figure 1:**
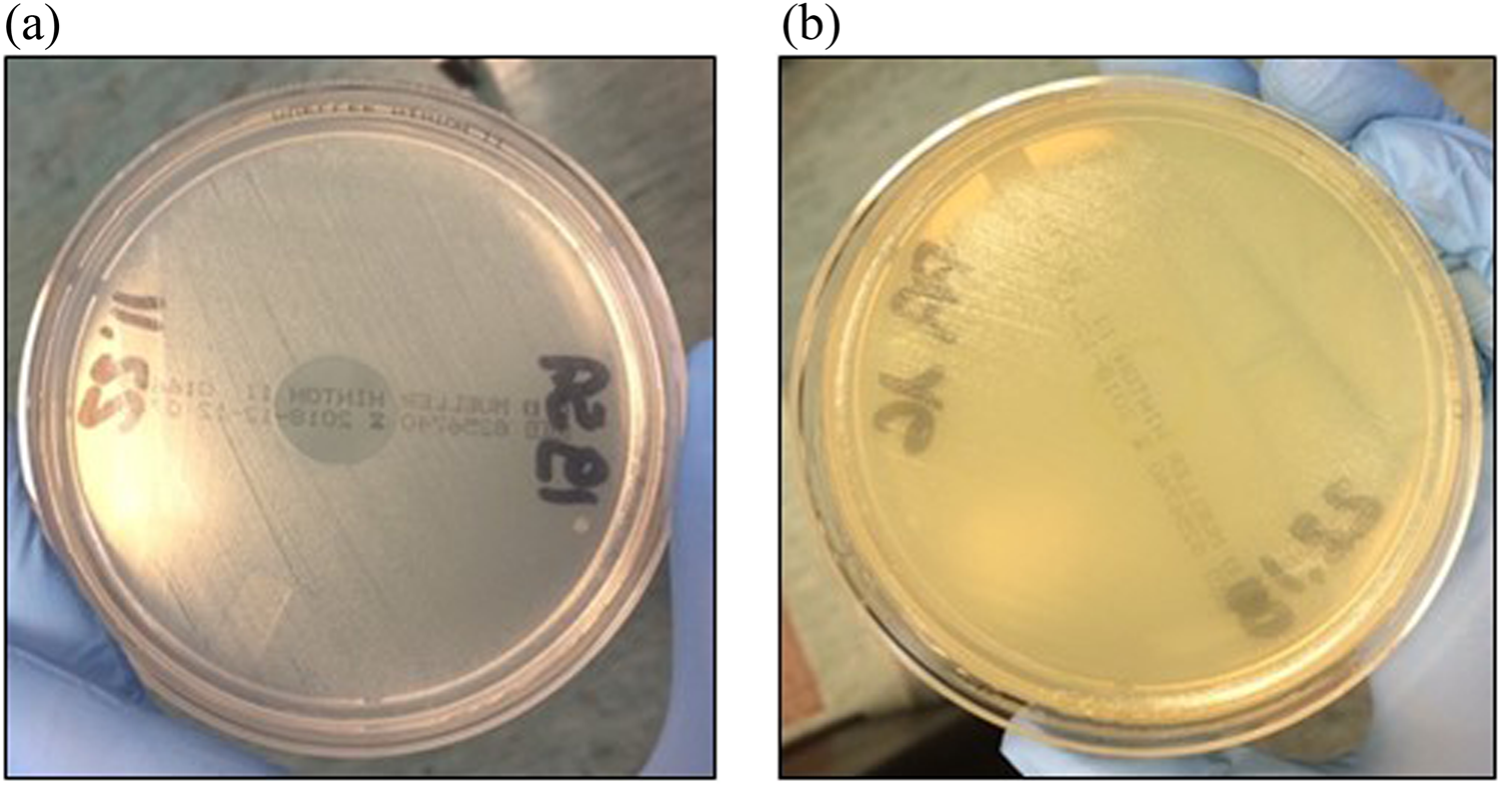
Culture of multidrug resistant *Pseudomonas aeruginosa.* (a) Bacterial culture after 6 h. An inhibition zone was observed at this time point. (b) Bacterial culture after 8 h. A recolonization in the area where the ozonated solution had been deposited was observed after 8 h.

**Figure 2:**
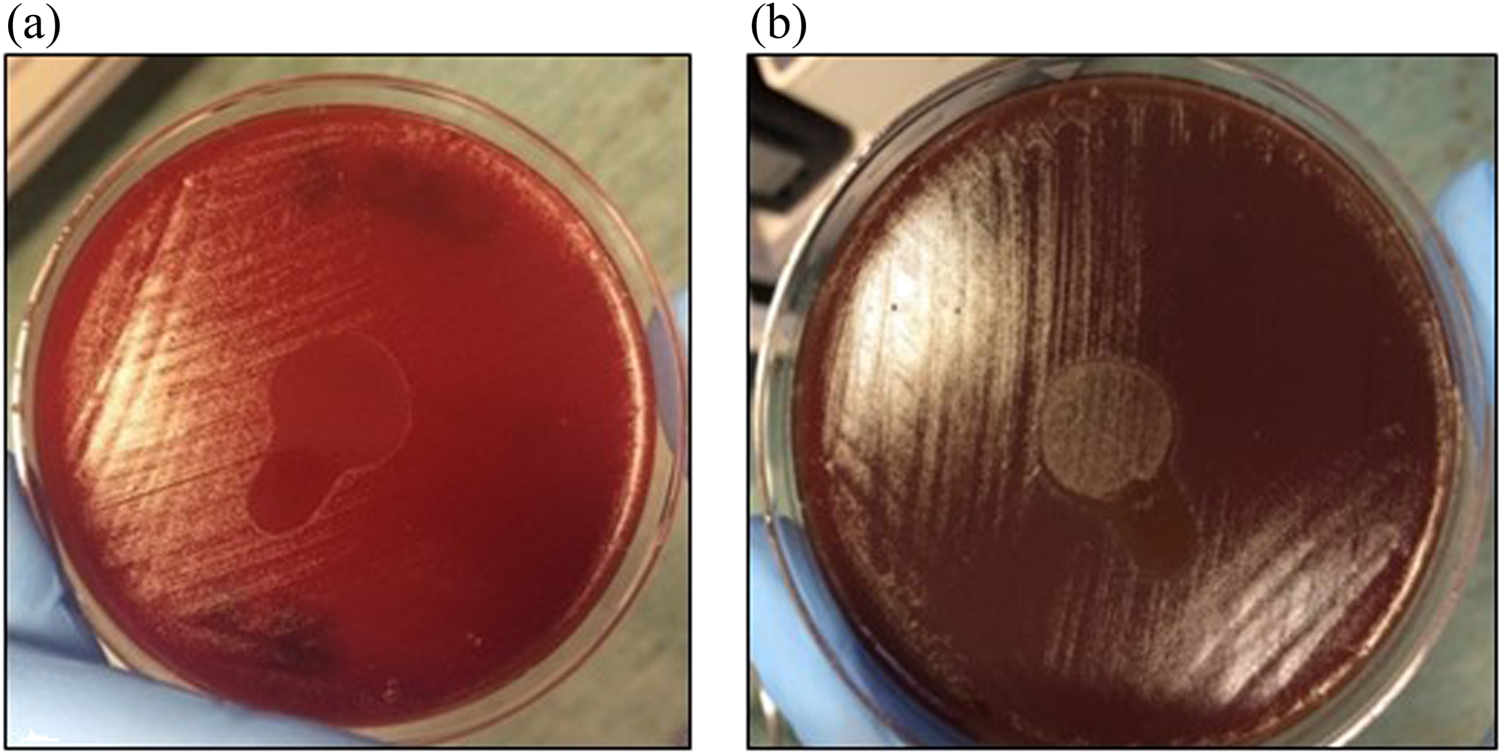
Inhibition zone of *Streptococcus agalactiae* strains after contact with the liposomal ozonated oil. (a) Inhibition zone after 6 h of contact with the liposomal ozonated oil. (b) Inhibition zone after 8 h of contact with the liposomal ozonated oil.

The inhibition zones of all isolates of the PA MDR strain were observed after 6 h (Figure 1a). At 8 h, 20% of the samples exhibited recolonization in the region where the ozonated solution had been deposited (Figure 1b). The PA MDR strains completely recovered the growth at 24 h. At 6 and 8 h, inhibition zones of the MRSA and MRSE strains were observed in the area around the ozonated oil, but not at 24 h (Table 1), suggesting that the potency of the oil to inhibit may decrease over time. Inhibition zones were observed in 100% of the *Streptococcus* spp. strains in the area that was in contact with the ozonated oil droplet after 6 and 8 h. However, after 24 h, the bacterial regrowth was inhibited in 70% of the *Streptococcus* spp. strains. In the remaining 30% of strains (all *S. agalactiae* strains), the growth was inhibited at 6 and 8 h, but not at 24 h (Figure 2a and b). Table 1 presents the changes observed in the bacterial cultures on the agar plates after the addition of the ozonated oil. At 8 h, 94% of all the strains tested showed an inhibition zone. After 24 h, the growth of more than 10% of the isolates was inhibited in the area of contact with the ozonated oil.

**Table 1:** Growth changes in the bacterial cultures after treatment with the liposomal ozonated oil

Microorganism	2–4 h incubation	6 h incubation	8 h incubation	24 h incubation
*Pseudomonas aeruginosa*	No visible inhibition	Inhibition zones	Inhibition zones but regrowth in 20% of strains	Regrowth
*Staphylococcus aureus*	No visible inhibition	Inhibition zones	Inhibition zones	Regrowth
*Staphylococcus epidermidis*	No visible inhibition	Inhibition zones	Inhibition zones	Regrowth
*Streptococcus* spp.	No visible inhibition	Inhibition zones	Inhibition zones	Regrowth in 30% of strains (all *S. agalactiae*)

## 4 Discussion

Ozone is a gaseous, triatomic allotrope of oxygen that has long been recognized as one of the most potent antibacterial, antiviral, and antifungal agents. This activity is a result of its high oxidation potential, which allows it to destroy the bacterial cell wall and cytoplasmic membrane, resulting in increased cell permeability, allowing the entry of ozone into the bacterial cell [37,38]. Furthermore, ozone is a powerful immunomodulator that can boost immune activity in the body. It can also oxidize lipids to produce hydrogen peroxide, which is also a strong antimicrobial agent. Oxygenated agents, such as ozonated water, vegetable oil, and animal fats, have been used to improve the short half-life of ozone. There are several parameters that characterize the quality of an ozonated oil: the type of oil, ozonization process applied, iodine value, acid value, peroxide value, viscosity, and absorption [39,40,41].

Ozonated olive oil has been used in the treatment of bacterial skin infections since the 1930s. Even today, ozonated oils are widely used in dermatology and dentistry to treat infected lesions [42]. This study aimed to verify the action of ozonated oil for topical ophthalmic use against MDR bacteria.

Song et al. evaluated the action of ozonated water and ozonated oil for topical dermatological use on methicillin-sensitive and methicillin-resistant strains of *S. aureus*. They demonstrated a 100% reduction in the bacterial load of both isolates after 15 min [43]. Also, Zanardi et al. used ozonated oil preparations to eliminate cutaneous infections. These authors reported a potent bactericidal effect of ozonated oils with a reduction of cutaneous infection exudates [44].

The present study is the first to test the effect of ozonated oil encapsulated in liposomes against MDR bacteria (MRSA, MRSE, and PA MDR) and *Streptococcus* spp., which are implicated in both superficial and deep eye diseases.

The ozonated solution exhibited antibacterial activity against all the tested species. The activity was lower against the PA MDR strains than against the other species and was more evident in *Staphylococcus* and *Streptococcus* spp. We believe that it was important to analyze multiple strains of each species, since it allowed us to highlight differences in the behavior of the solution, not only toward the different genera and species but also toward individual strains, given that after 8 h, not all strains of PA MDR recolonized the zone of inhibition, and after 24 h, not all *Streptococcus* species were inhibited (only *S. agalactiae*, a rare pathogen in eye infection). This finding is particularly significant because the mechanism of antimicrobial action of ozonated oil appears to be nonspecific and nonselective. Therefore, in theory, we would not expect any differences in activity against the bacterial strains. Further studies are necessary to investigate and explain these observations.

Another interesting aspect is the duration of bacterial growth inhibition by the ozonated oil, which was found to be 6 h for all species and strains, with regrowth in 20% of the PA MDR strains after 8 h and 30% of *Streptococcus* spp. after 24 h. These observations allow us to define an *in vivo* drug administration approach, both empirically and when the pathogen is unknown. Therefore, the administration of ozonated oil every 6–8 h, as in the clinical practice, should completely inhibit even the most resistant bacterial species, whether endogenous to the ocular flora like *S. aureus* and CoNS, or exogenous like *P. aeruginosa*. However, this hypothesis should be confirmed *in vivo*.

Interestingly, liposomal ozonated oil has long-lasting effects on *Streptococcus* spp., which are responsible for a large proportion of ocular infections.

From the data collected in this study, it can be concluded that the ozonated oil has an inhibitory and therefore bacteriostatic effect on MDR bacteria. However, it is necessary to assess if it also possesses bactericidal properties. Re-colonization may be a result of the bacterial growth outside the zone of inhibition, while those originally present in the zone are killed (bactericidal effect). Alternatively, only the growth of bacteria present in the zone of inhibition may be inhibited (bacteriostatic effect). Bactericidal properties could make the action of the product even more effective.

With the exception of *Streptococcus* spp., all MDR strains that were selected were very difficult to treat with antibiotics. It is clear from the literature that the incorrect and unwarranted use of antibiotic eye drops has resulted in the emergence of MDR strains, meaning that even infections that appear to be trivial can become difficult to treat. According to the principles of evidence-based medicine, it is type A evidence that in the majority of cases of conjunctivitis (suspected or confirmed), without other ocular and/or systemic comorbidities, antibiotic treatment is not necessary since the condition is self-limiting and generally resolves within 7–10 days. However, it is also type A evidence that the use of antibiotics can reduce the duration of the disease, allowing faster reintegration into work or school. Therefore, the administration of broad-spectrum antibiotics to treat bacterial conjunctivitis may be considered reasonable.

There is a significant controversy around empirical topical therapy, and references are often made to the local pattern of resistance, cost, dosage, and other patient-related factors, such as allergies and compliance [45]. Therefore, it is possible to understand how the inappropriate use of antibiotics in a self-limiting and widespread pathology can potentially cause the emergence of bacterial resistance. Therefore, antiseptics can be extremely valuable, providing at least partial protection against the development of resistance, while also shortening the course of the disease and allowing patients to return to their normal activities.

The increase in bacterial resistance, including bacteria that cause ocular pathologies, has been demonstrated by the “Ocular Tracking Resistance in U.S. Today” Study (TRUST) [23] and the “Antibiotic Resistance Among Ocular Pathogens in the United States” study (ARMOR) [24,26]. This becomes an even more serious problem in the case of infections of the anterior and posterior segments of the eye and in preoperative prophylaxis. Therefore, the search for new antibacterial compounds capable of preventing the onset of resistance has become increasingly important. The possibility of using ozonated oil in liposomes has already been demonstrated in an animal model. However, this study was limited to coagulase-positive and coagulase-negative staphylococci (34). A more in-depth study on the bactericidal effect of ozonated oil encapsulated in liposomes will have to be carried out to evaluate its full potential, including the evaluation of its oxidative effect on the ocular surface, which is particularly delicate and unique. The data from this study cannot be compared with those from other studies reported in the literature, since the only other study that evaluated the efficacy of an ozonated product against MDR bacteria applied a different methodology. Moreover, a previous study assessed an oil for skin application, as opposed to an ophthalmic product. The ionization characteristics of the two ozonated lipids were also not known for comparison.

A key limitation of our study is largely related to its qualitative nature, thus limiting the applicability in clinical practice. Another clear limitation of our study is that we performed an *in vitro* experiment, which may not reflect exactly the real situation *in vivo*.

We believe that in-depth research into the characteristics of the oil and its ozonation may be the key to optimizing and improving antimicrobial therapy. The use of ozonated oil in ophthalmology, such as in preoperative prophylaxis, can be useful, as antibiotics are now likely to fail or lead to the development of new drug resistance. This approach to therapy can broaden the spectrum of action and prevent the emergence of drug-resistant strains. The possibility of providing clinicians with a new tool, as an alternative to antibiotics, can be fundamental in bacterial endophthalmitis prophylaxis for cataract surgery and intravitreal treatment and in improving therapeutic strategies, broadening the antibiotic spectrum, and avoiding viral and fungal superinfections.
